# The role of Rab6a and phosphorylation of non-muscle myosin IIA tailpiece in alcohol-induced Golgi disorganization

**DOI:** 10.1038/srep31962

**Published:** 2016-08-18

**Authors:** Armen Petrosyan, Carol A. Casey, Pi-Wan Cheng

**Affiliations:** 1 Department of Biochemistry and Molecular Biology, College of Medicine, the Fred and Pamela Buffett Cancer Center, 985870 Nebraska Medical Center, Omaha, NE 68198-5870, USA; 2 Department of Internal Medicine, University of Nebraska Medical Center, Omaha, NE, USA; 3 Nebraska Western Iowa Health Care System, VA Service, Department of Research Service, Omaha, NE, USA

## Abstract

Abnormalities in the Golgi apparatus function are important to the development of alcoholic liver injury. We recently reported that Golgi disorganization in ethanol (EtOH)-treated hepatocytes is caused by impaired dimerization of the largest Golgi matrix protein, giantin. However, little is known about the mechanism which forces fragmentation. Here, in both HepG2 cells overexpressing alcohol dehydrogenase and in rat hepatocytes, we found that EtOH administration reduces the complex between giantin and Rab6a GTPase and results in the S1943 phosphorylation of non-muscle Myosin IIA (NMIIA) heavy chain, thus facilitating NMIIA association with Golgi enzymes, as detected by biochemical approaches and 3D Structured Illumination Microscopy. We revealed that NMIIA-P-S1943 competes with giantin for the Rab6a dimer, which was converted to monomer after Golgi fragmentation. Therefore, Rab6a plays a dual role in the Golgi, serving as master regulator of Golgi organization and disorganization, and that NMIIA and giantin engage in a “tug-of-war”. However, the inhibition of F-actin and downregulation of NMIIA or overexpression of NMHC-IIAΔtailpiece, as well the overexpression of dominant negative Rab6a(T27N), preserved a compact Golgi phenotype. Thus, the actomyosin complex forces EtOH-induced Golgi disorganization, and the targeting of NMIIA-P-S1943 may be important for preventing the damaging effects of alcohol metabolism on the cell.

Chronic alcohol abuse and alcoholism are associated with high morbidity and mortality; they are known to cause alcoholic liver disease (ALD), myocardial infarction, pancreatitis, and disorders of the immune, endocrine, and reproductive systems^1^. In 2011, liver cirrhosis was the 12th leading cause of death in the United States. Of these deaths, 48% were ALD-related^2^. It is known that chronic and acute ethanol (EtOH) consumption is associated with hepatocellular steatosis and necrosis, the starting point of ALD. The grand effect of alcohol metabolism’s damaging effects on the cell is notably linked to alteration in the structure of intracellular membranes, including the Golgi apparatus^3^,^4^,^5^,^6^,^7^. We have recently shown that alcohol dehydrogenase (ADH)-catalyzed oxidation of EtOH is a major contributor to Golgi fragmentation, and that this fragmentation is initiated by downregulation of Sar1a GTPase, an essential component of the COPII vesicle^8^. Under normal conditions, these vesicles are responsible for Golgi delivery of the protein disulfide isomerase A3 (PDIA3), an enzyme that catalyzes dimerization of giantin, the largest Golgi matrix protein. EtOH treatment impairs formation of COPII, arrests PDIA3 in the endoplasmic reticulum (ER), and prevents the subsequent dimerization of giantin. In consequence, Golgi loses its intercisternal connection and appears disorganized. These data mirror a concept that emerged recently, namely, that giantin integrity is necessary for maintaining normal Golgi structure^9^,^10^,^11^. Thus, the dysfunction of Golgi matrix proteins is one of the main contributors of EtOH-induced Golgi scattering; however, to be separated into multiple structures, Golgi membranes require the assistance of motor proteins, which are known to control tension-driven flow^12^.

Recent observations clearly indicate that among other motor proteins closely associated with Golgi, the non-muscle Myosin IIA (NMIIA) interacts directly with glycosyltransferases (GTs), the Golgi resident proteins^13^. We and others have shown that NMIIA is tethered to the Golgi membranes under different stress conditions, such as treatment with Brefeldin A, heat shock or inhibition of heat shock proteins (HSPs), and depletion of beta-COP^14^,^15^,^16^. Depletion or inhibition of NMIIA also prevents Golgi disorganization and restores the compact Golgi morphology in prostate and colon cancer cells^11^,^15^. In light of this, it is attractive to speculate that NMIIA is the universal regulator of Golgi remodeling. In turn, this prompted us to ask whether EtOH-induced Golgi fragmentation is also mediated by NMIIA, and whether this action requires involvement of Rab6a, a small GTPase known to interact with both NMIIA and giantin^17^,^18^.

Here, the chemical inhibition or depletion of NMIIA demonstrates that this F-actin-based motor protein governs EtOH-induced Golgi fragmentation. While Rab6a-Giantin cooperation provides Golgi integrity, the complex between Rab6a and NMIIA controls Golgi disorganization. We found that structural organization of Golgi in hepatocytes is modulated by sequential interaction of Rab6a with Giantin and NMIIA. EtOH treatment induces phosphorylation of NMIIA at S1943 (NMIIA-P-S1943). NMIIA-P-S1943, in turn, competes with Giantin for Rab6a, and thus facilitates its interaction with Golgi membranes, likely through cooperation with Golgi residential enzymes.

## Results

### EtOH-induced Golgi fragmentation is mediated by NMIIA and F-actin

The alteration of tubulins has been reported, particularly acetylation by acetaldehyde, the primary metabolite of EtOH^19^,^20^. However, these modified microtubules are more resistant to depolymerization induced by Nocodazole^21^. Given that Nocodazole induces Golgi scattering^15^, these data suggest that the microtubule remodeling that is induced by EtOH is not the cause of Golgi fragmentation. On the other hand, EtOH does not alter actin filament organization or its polymerization^22^, implying that the Golgi-to-ER transport is not affected by EtOH. Because this trafficking is mediated by NMIIA^13^,^14^, and NMIIA is abundant in the human liver^23^, we analyzed whether ablation of NMIIA function alleviates Golgi disorganization induced by EtOH treatment.

**Figure 1:**
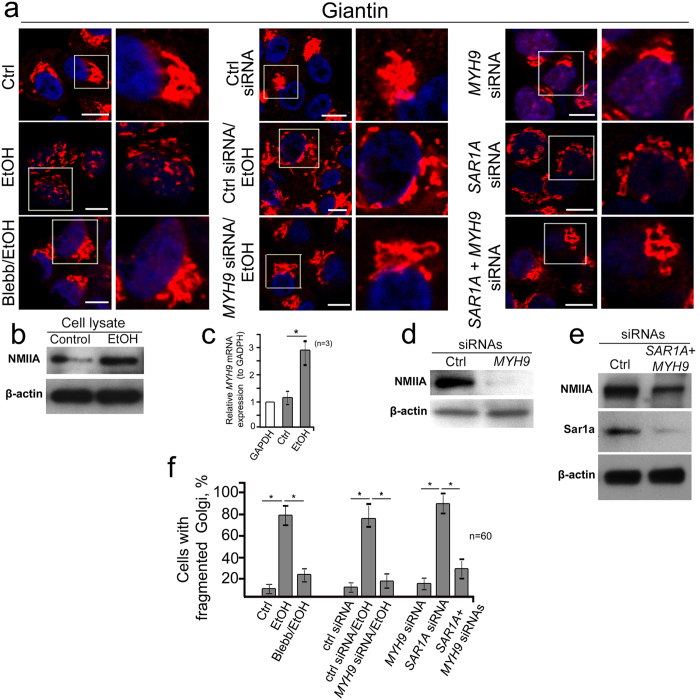
Non-muscle Myosin IIA is the governor of EtOH- and Sar1a-KD-induced Golgi fragmentation. (**a**) Immunostaining of giantin in VA-13 cells (HepG2 cells expressing ADH). First row: no treatment (Ctrl), EtOH treatment (35 mM for 72 h) and EtOH treatment followed by 25 μM blebbistatin at 48 h; second row: control siRNA, and 35 mM EtOH for 72 h plus control or *MYH9* siRNAs; third row: *MYH9* siRNAs, *SAR1A* siRNAs or *SAR1A*+*MYH9* siRNAs. The right panel shows high magnifications of the highlighted area (white boxes). Nuclei were counterstained with DAPI (blue). (**b**) NMIIA Western blot of the lysates of VA-13 cells treated with 35 mM EtOH for 72 h; β-actin was a loading control. (**c**) Quantitative real-time PCR analysis of the mRNA of NMIIA in control VA-13 cells and treated with 35 mM EtOH for 72 h. Data were presented as a mean from the three independent experiments and expressed as the fold relative to that (100%, 1 fold) of GAPDH. (**d**) NMIIA Western blot of the lysates of VA-13 cells treated with scramble or *MYH9* siRNAs; β-actin was a loading control. (**e**) NMIIA and Sar1a Western blot of the lysates of VA-13 cells treated with scramble or S*AR1A* plus *NMIIA* siRNAs; β-actin was a loading control. (**f**) Quantification of the fragmented Golgi in cells treated as described in the (**a**) n = 60 cells from three independent experiments.

As we previously reported, treatment of VA-13 cells (HepG2 cells overexpressing ADH^8^,^24^) with 35 mM EtOH for 72 h changes Golgi morphology from a compact structure localized at the perinuclear space to multiple fragmented bodies (Fig. 1a, first row). At the same time, the level of NMIIA protein and its mRNA expression were elevated (Fig. 1b,c), which showed agreement with our previous observations that NMIIA-mediated Golgi fragmentation is accompanied by its elevation^15^,^16^. To validate this screening, we performed chemical inhibition of NMIIA by treatment with selective myosin II inhibitor blebbistatin (Blebb)^25^. Because the Golgi morphological changes appeared after 48 h treatment with EtOH^8^, we treated VA-13 cells with 35 mM EtOH for 48 h followed by 35 mM EtOH plus 25 μM of Blebb for another 24 h. As shown in Fig. 1a (first row) and f, the number of cells with fragmented Golgi were identical in control cells and in cells treated with EtOH plus Blebb, but were significantly low compared to those treated with only EtOH. In support of these data, we further observed that simultaneous treatment of VA-13 cells with NMIIA (*MYH9*) siRNAs (Fig. 1d) and EtOH prevents fragmentation of the Golgi, because the number of cells with disorganized Golgi were similar to the cells treated with either control or NMIIA siRNA (Fig. 1a, second and third row, and f). Because the downregulation of Sar1a GTPase in EtOH-treated hepatocytes was interpreted as a key event initiating Golgi fragmentation^8^, we investigated the effect of Sar1a and NMIIA co-knockdown (KD) on Golgi morphology. Whereas *SAR1A* silencing results in Golgi fission^8^,^26^,^27^,^28^, Sar1a+NMIIA depletion preserves compact Golgi morphology (Fig. 1a, third row, e and f), indicating that Golgi fragmentation induced by both EtOH and Sar1a siRNA treatment is triggered by the same mechanism.

**Figure 2:**
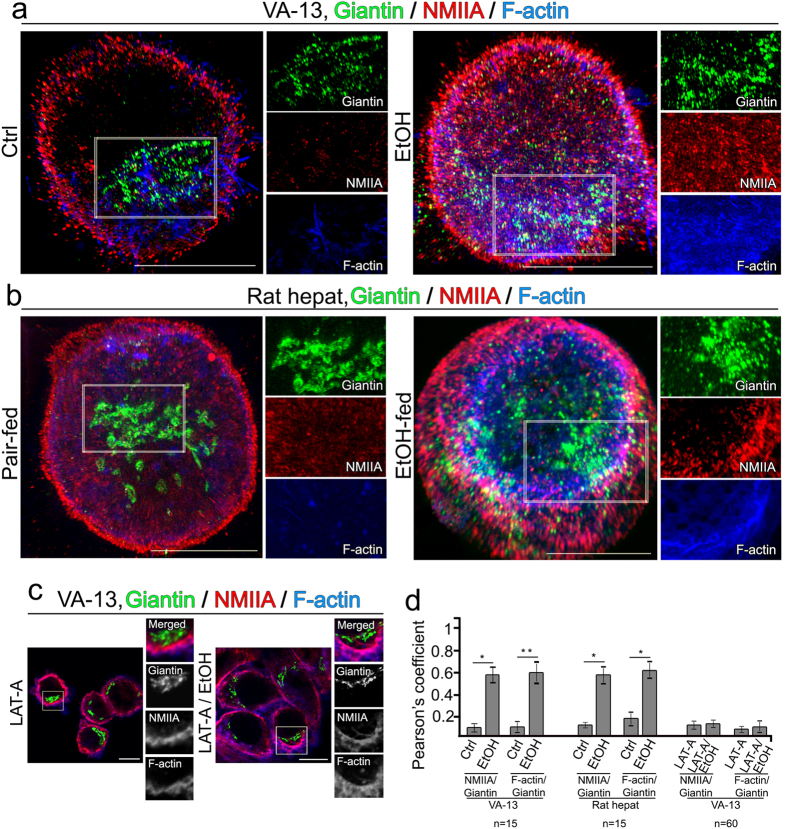
EtOH-induced Golgi fission is prevented by inhibition of F-actin. (**a,b**). Representative 3D SIM imaging of VA-13 cells: (**a**) control and treated with 35 mM EtOH for 72 h; (**b**) hepatocytes: from control and EtOH-fed rats. Cells were immunostained for giantin (green), NMIIA (red) and stained for F-actin by blue fluorescent phalloidin conjugate. The right panel shows high magnifications of green, red and blue channels corresponding to the perinuclear space (white boxes). (**c**) VA-13 cells treated with 0.2 μM Latrunculin A (LAT-A) for 24 h and EtOH treatment followed by LAT-A at 48 h. Cells were immunostained for giantin (green), NMIIA (red) and stained for F-actin by blue fluorescent phalloidin conjugate. The right panel shows high magnifications of merged, green, red and blue channels corresponding to the perinuclear space (white boxes). Merged channel is presented in color, while others in black-and-white. All confocal images were acquired with the same imaging parameters; bars, 10 μm. (**d**) Quantification of Pearson’s coefficient between Golgi-associated NMIIA, F-actin and giantin in cells presented in (**a**–**c)**, respectively. Results are expressed as a mean ± SD from three independent experiments; *p < 0.001, **p < 0.01.

Because the contractile function of NMIIA is provided by F-actin^29^, we tested whether EtOH-induced Golgi fission would be prevented by latrunculin A (LAT-A), an inhibitor of F-actin assembly^30^. For this purpose, we employed structured illumination super-resolution microscopy (SIM) which allowed us to achieve two-color 3D imaging at ~110 nm resolution. Using F-actin labeling kit-blue fluorescence, we detected in control VA-13 cells and in hepatocytes isolated from control rats a marginal fraction of Golgi-specific F-actin- and NMIIA-positive punctae, as measured by Pearson’s coefficient between these two proteins and giantin (Fig. 2a,b, Ctrl and d). In EtOH-treated VA-13 cells and hepatocytes isolated from EtOH-fed rats, the colocalization points were detected across a much higher number of Golgi objects (2a, b, EtOH and d). LAT-A treatment had no significant effect on Golgi morphology, except the induction of a mild compaction^31^ (Fig. 2c, LAT-A). In cells co-treated with EtOH plus LAT-A, we observed a segregation of both F-actin and NMIIA from Golgi, but not Golgi disorganization (Fig. 2c, LAT-A/EtOH, and d), suggesting that both NMIIA and F-actin are necessary for EtOH-induced Golgi fragmentation.

### Rab6a GTPase is an essential regulator of EtOH-induced Golgi fission

**Figure 3:**
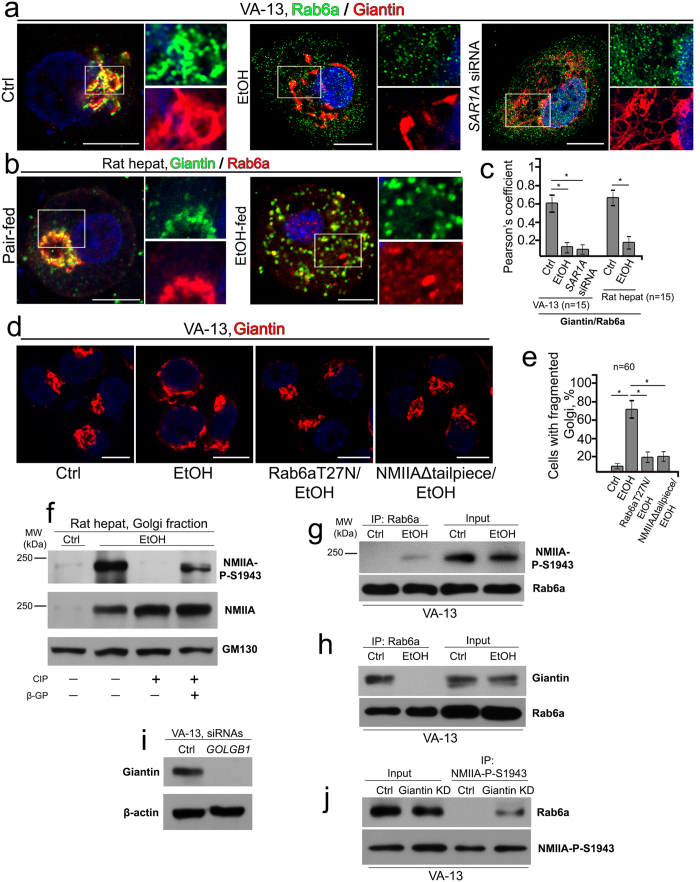
EtOH-induced Golgi fragmentation is regulated by Rab6a GTPase. (**a,b**) Immunostaining of Rab6a (green) and giantin (red) in (**a**) VA-13 cells: control, EtOH- or *SAR1A* siRNA-treated; (**b**) immunostaining of giantin (green) and Rab6a (red) in hepatocytes isolated from control and EtOH-fed rats. The right panels show green and red channels corresponding to Golgi and cytoplasmic regions (white boxes). Nuclei were counterstained with DAPI (blue). All confocal images were acquired with same imaging parameters; bars, 10 μm. (**c**) Quantification summarizing the Rab6a-specific fluorescence signal colocalized with giantin in cells presented in (**a**,**b**). The Pearson’s coefficient is presented as a mean ± SD from three independent experiments; *p < 0.001. (**d**) Immunostaining of giantin in VA-13 cells transiently transfected with empty PCMV vector (Ctrl), treated with 35 mM EtOH for 72 h and simultaneously transiently transfected with empty PCMV vector (EtOH), or transfected with Rab6(T27N) or NMIIAΔtailpiece plasmids. (**e**) Quantification of the fragmented Golgi in cells treated as described in the (**d**) n = 60 cells from three independent experiments. (**f**) NMIIA-P-S1943 Western blot of the Golgi fraction isolated from hepatocytes of control and EtOH-fed rats. Golgi membranes were isolated from cells as described in Methods and normalized by the total protein. EtOH sample was treated with CIP in the presence or absence of β-GP. (**g,h**) NMIIA-P-S1943 and Rab6a (**g**), and giantin and Rab6a (**h**) Western blot of the complexes pulled down with Rab6a Ab from the lysate of control VA-13 cells or cells treated with 35 mM EtOH for 72 h. Input was normalized by NMIIA-P-S1943 (**g**) or giantin (**h**). (**i**) Giantin Western blot of the lysates of VA-13 cells treated with scramble or *GOLGB1* (giantin) siRNAs; β-actin was a loading control. (**j**) Rab6a and NMIIA-P-S1943 Western blot of the complexes pulled down with NMIIA-P-S1943 Ab VA-13 cells treated with scramble or *GOLGB1* (giantin) siRNAs; input was normalized by Rab6a.

We previously demonstrated in prostate cancer cells that the Golgi-specific GTPase Rab6a is involved in fragmentation of the Golgi through cooperation with NMIIA^11^. Here, we wondered if alcohol-specific Golgi fragmentation might be mediated by sequential interaction of Rab6a with giantin and NMIIA, because the complex of Rab6a and NMIIA has been shown to be involved in Golgi remodeling and the extension of Golgi tubules to the ER^18^,^32^. To analyze the effect of EtOH on Rab6a distribution, we quantified a Golgi-specific signal in both VA-13 cells and rat hepatocytes by measuring Rab6a co-staining with giantin. In control cells, Rab6a was strongly colocalized with giantin, as judged by Pearson’s coefficient - 0.61 and 0.69 in VA-13 cells and rat hepatocytes, respectively (Fig. 3a–c). In contrast, in response to EtOH treatment, Rab6a colocalization with giantin was reduced to 0.13 in VA-13 cells and 0.18 in rat hepatocytes (Fig. 3a–c). It is also important to note that the distribution of Rab6a in VA-13 cells lacking Sar1a (after *SAR1A* siRNA treatment) was similar to that in cells treated with EtOH (Fig. 3a,c), suggesting that, analogously to the EtOH treatment, Rab6a is partially segregated from giantin after depletion of Sar1a. Furthermore, transiently transfected cells over-expressing dominant negative (GDP-bound) Rab6(T27N) are able to maintain compact Golgi despite EtOH treatment, confirming that in EtOH-treated hepatocytes Rab6a regulates NMIIA-mediated Golgi fragmentation (Fig. 3d,e).

### EtOH treatment induces S1943 phosphorylation of NMIIA

The association of NMIIA with Golgi membranes and Golgi-derived vesicles was detected by many different groups^13^,^18^,^33^,^34^,^35^,^36^, with most authors unanimous in stating that NMIIA is connected to Golgi through its C-terminal region. Of importance, the C-terminal non-helical tailpiece (1928–1960 aa) of NMIIA determines the intracellular localization of NMIIA^37^, and this prompted us to examine if this last C-terminal segment plays a significant role in tethering NMIIA to the Golgi. As shown in Fig. 3d,e, when VA-13 cells were transfected with an NMIIA mutant lacking the last 33 amino acids (corresponding to the non-helical tailpiece), followed by treatment with EtOH, the number of cells with fragmented Golgi was significantly reduced, validating the importance of the tailpiece for EtOH-induced Golgi disorganization. It is known that NMIIA is able to bind to the Golgi as a dimer but not as typical, large bipolar filaments^35^. Given that Ser1943 in the non-helical NMHC tail is a substrate for phosphorylation by casein kinase 2 (CK II), and S1943 NMIIA phosphorylation inhibits the assembly of NMIIA rods into filaments^38^,^39^, it is logical to assume that NMIIA-P-S1943 is involved in EtOH-induced Golgi fragmentation. Whereas in control hepatocytes we could barely detect NMIIA-P-S1943 in the Golgi membranes fraction, after EtOH treatment, the level was greatly elevated (Fig. 3f). Further, an NMIIA-P-S1943-specific band was resolved after treatment of Golgi fraction with calf intestinal alkaline phosphatase (CIP). Thereafter, the phosphatase-specific action was abolished after treatment with a general phosphatase inhibitor, β-glycerophosphate (β-GP) (Fig. 3f and Supplementary Fig. S1). With this background, we concluded that the increase of giantin-independent Rab6a-positive punctae in EtOH-treated samples (Fig. 3a–c) could be partially due to increased association of Rab6a with NMIIA-P-S1943. To test this, we performed an IP analysis, which showed that Rab6a and NMIIA-P-S1943 were present in the same complex in EtOH-treated but not in control VA-13 cells (Fig. 3g). Conversely, and contrary to the control cells^17^, little if any fraction of giantin was detected in Rab6a IP samples from EtOH-treated cells (Fig. 3h and Supplementary Fig. S2). Finally, to prove that the enhanced cooperation of Rab6a with NMIIA is a direct consequence of EtOH-induced deficiency of giantin, we performed in VA-13 cells KD of giantin using *GOLGB1* (giantin) siRNAs (Fig. 3i and Supplementary Fig. S3). Then, cell lysate was subjected to the NMIIA-P-S1943 IP followed by Western blot probed with anti-Rab6a Ab. As shown in Fig. 3j and Supplementary Fig. S4, no complex between Rab6a and NMIIA-P-S1943 was detected in cells treated with scrambled siRNA, while in cells lacking giantin (after treatment with *GOLGB1* siRNAs) their interaction was evident.

### NMIIA-P-S1943 interacts with Golgi glycosyltransferases

**Figure 4:**
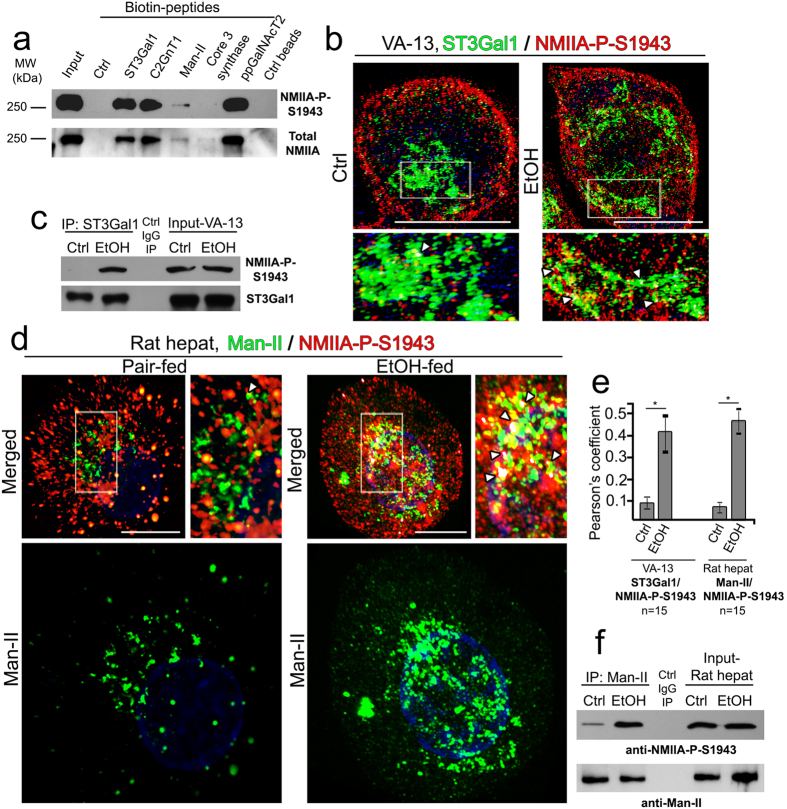
NMIIA-P-S1943 associates with Golgi glycosyltransferases. (**a**) NMIIA-P-S1943 and total NMIIA Western blot of the complexes pulled down from the lysates of EtOH-treated VA-13 cells with biotinylated control peptide, human ST3Gal1, C2GnT1, Man-II, core 3 synthase, and ppGalNAcT2 N-terminal peptides, cell lysate fraction isolated by magnetic beads without peptides, and cell lysate. (**b**) Representative 3D SIM imaging of VA-13 cells labeled with ST3Gal1 (green) and NMIIA-P-S1943 (red) before and after EtOH treatment. The Golgi area in the white box is enlarged and presented as merged channel below. (**c**) NMIIA-P-S1943 and ST3Gal1 Western blot of the complexes pulled down with anti-ST3Gal1 Ab from the lysate of control or EtOH-treated VA-13 cells. (**d**) Representative 3D SIM imaging of control rat hepatocytes and hepatocytes isolated from EtOH-fed rats and labeled with Man-II (green) and NMIIA-P-S1943 (red). The Golgi area in the white box is enlarged and presented as a merged on the right; the green channel is presented below. Colocalization area in (**b**,**d**) (white spots) indicated by white arrowheads were analyzed with the automatic threshold method of Imaris Coloc (Imaris software); bars, 10 μm. (**e**) Quantification of Pearson’s coefficient in cells presented in (**b**,**d**), respectively; n = 15 cells from three independent experiments; *p < 0.001. (**f**) NMIIA-P-S1943 and Man-II Western blot of the complexes pulled down with anti-Man-II Ab from the lysate of hepatocytes from control or EtOH-fed rats.

Consistent with our previous observations^13^,^15^, we hypothesize that NMIIA-P-S1943 uses the cytoplasmic tail of Golgi GTs as the anchor to tether to Golgi membranes. To determine if NMIIA-P-S1943 directly interact with Golgi enzymes, we isolated NMIIA-P-S1943 with biotinylated peptides representing the cytoplasmic tail of different Golgi residential proteins: β-galactoside α-2,3-sialyltransferase-1 (ST3Gal1), core 2N-acetylglucosaminyltransferase-1 (C2GnT1), β1,3-N-acetylglucosaminyltransferase 6 (core 3 synthase), α-mannosidase II (Man-II), polypeptide N-acetylgalactosaminyltransferase 2 (ppGalNAcT2). The level of pulled-down NMIIA-P-S1943 varied considerably between samples, indicating the different binding affinity of these cytoplasmic tail peptides with NMIIA-P-S1943; however, NMIIA-P-S1943 was not detected either in control beads exposed to cell lysate or in sample with control peptide (Fig. 4a, top panel and Supplementary Fig. S5). Importantly, we found that the differences in the intensity of bands were identical for both NMIIA-P-S1943 and total NMIIA (Fig. 4a, low panel and Supplementary Fig. S5), suggesting that the observed bands are likely belong to the NMIIA-P-S1943. To further examine the association of NMIIA-P-S1943 with Golgi GTs, we employed SIM microscopy; specifically, in VA-13 cells, we monitored colocalization of NMIIA-P-S1943 with ST3Gal1. As shown in Fig. 4b,e, in control cells the ST3Gal1 signal was predictably detected in the compact Golgi structure, and displayed only minor overlap with NMIIA-P-S1943; however, in EtOH-treated cells, ST3Gal1 puncta were distributed throughout the cell, and NMIIA-P-S1943 displayed multiple colocalization puncta (white arrowheads) (Supplemental movie 1 and 2). In VA-13 cells treated with EtOH we were consistently able to pull down more NMIIA-P-S1943 using IP with ST3Gal1 Ab, than that in untreated cells (Fig. 4c and Supplementary Fig. S6). Similarly, in control rat hepatocytes, Man-II puncta were concentrated in the perinuclear area and mostly segregated from NMIIA-P-S1943, but in hepatocytes from EtOH-fed rats, NMIIA-P-S1943 was abundantly detected at fragmented Man-II-stained structures (Fig. 4d,e, white arrowheads) (Supplemental movie 3 and 4). Their interaction was further confirmed by IP experiment: more NMIIA-P-S1943 was pulled down by Man-II Ab from the lysate of hepatocytes from EtOH-fed rats (Fig. 4f and Supplementary Fig. S7) in comparison with control samples.

### NMIIA-P-S1943 competes with giantin for Rab6a

**Figure 5:**
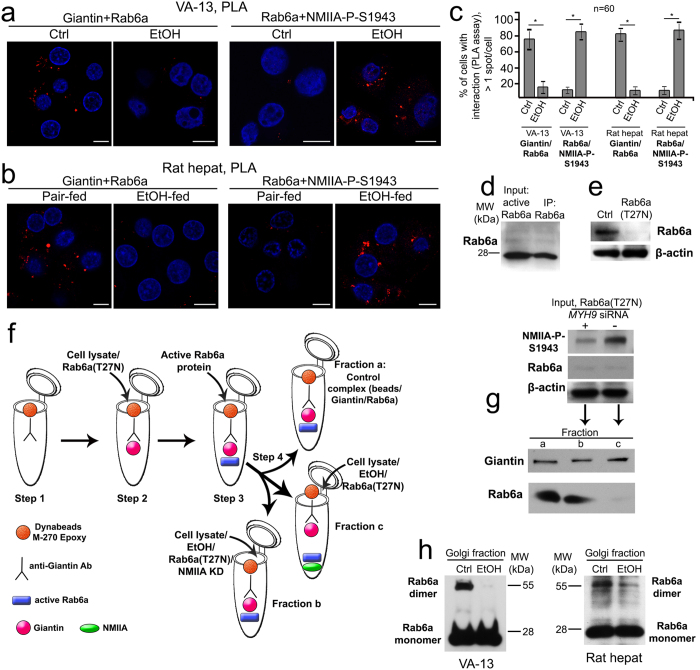
EtOH effect on Rab6a: the loss of dimer form, reduction of interaction with giantin, and enhancement of association with NMIIA. (**a,b**) Proximity ligation assay (PLA) of Giantin/Rab6a and Rab6a/NMIIA-P-S1943 in control VA-13 cells and treated with 35 mM EtOH for 72 h (**a**), and in hepatocytes from control and EtOH-fed rats (**b**) as analyzed by confocal microscopy; bars, 10 μm. (**c**) Quantification of PLA red spots (close proximity sites) scored per cell with more than one spots; n = 60 cells. The results are presented as a mean ± SD from three independent experiments; *p < 0.001. (**d**) Rab6a Western blot of the human active Rab6a recombinant protein and its IP with anti-Rab6a Ab. One μg of Rab6a protein was used as an input. (**e**) Rab6a Western blot of the cell lysate of VA-13 cells transiently transfected with empty PCMV vector (Ctrl) or with Rab6(T27N) plasmid; β-actin was a loading control. (**f**) Schema of the *in vitro* Rab6a capturing experiment. Anti-giantin-Ab was exposed to Dynabeads M-270 (step 1) followed by incubation with cell lysate of VA-13 cells overexpressing Rab6aT27N (step 2) and active Rab6a protein (step 3). The beads containing the complex anti-Giantin Ab-Giantin-Rab6a were: served as a control (fraction *a*), incubated with cell lysate of VA-13 cells overexpressing Rab6aT27N and treated with NMIIA siRNAs (fraction *b*), and incubated with cell lysate of VA-13 cells overexpressing Rab6aT27N (fraction *c*). (**g**) Giantin and Rab6a Western blot of the fractions *a*, *b*, and *c*, as described in f. Top panel indicates the input for fractions *b* and *c*. (**h**) Rab6a Western blot of the Golgi fraction from control and EtOH-treated VA-13 cells, and hepatocytes from control and EtOH-fed rats. The results shown are representative of three independent experiments.

In sum, these data suggest that in response to EtOH treatment, Rab6a loses interaction with giantin, but acquires a new association with NMIIA. This was tested using the proximity ligation assay (PLA) to monitor the dynamic of proximity of NMIIA-P-S1943 and Rab6a after EtOH treatment. Red staining is considered positive for close proximity between two target proteins^40^. We counted cells with more than one red spot and found that in around 78% of control VA-13 cells and 83% of rat hepatocytes, giantin and Rab6a (detected by anti-mouse giantin and anti-rabbit Rab6a, respectively) are present in a complex, whereas in cells treated with EtOH the positive signal was greatly reduced (Fig. 5a–c). In contrast, in control cells weak signal was obtained using anti-mouse Rab6a and anti-rabbit NMIIA-P-S1943, while in EtOH-treated samples the percentage of cells with PLA-positive puncta was enhanced to around 80% in VA-13 cells and 86% in rat hepatocytes (Fig. 5a–c). Thus, EtOH treatment appears to diminish the binding of giantin and Rab6a, which prompted us to hypothesize that NMIIA-P-S1943 competes with giantin for Rab6a.

We then performed a proof-of-principle experiment using human, full-length, active Rab6a protein, and of note, anti-Rab6a Ab successfully pulled down this Rab6a protein (Fig. 5d). Also, we found that the wild-type Rab6a was reduced in cells overexpressing Rab6(T27N) (Fig. 5e). First, we coupled anti-giantin-Ab to Dynabeads M-270 Epoxy using Dynabeads Antibody coupling kit (Life Science Technologies) according to the manufacture’s instruction (Fig. 5f, step 1). Then, to eliminate the contribution of endogenous Rab6a to the formation of complexes with NMIIA or giantin, we transiently transfected VA-13 cells with Rab6(T27N). The obtained cell lysate was incubated with Dynabeads, exposing giantin Ab at 4 °C for 12 h (Fig. 5f, step 2), followed by the addition of active Rab6a protein (5 μg) at 4 °C for another 12 h (Fig. 5f, step 3). After thorough washing in PBS, beads were isolated by the Magnetic Separation Rack (New England BioLabs) and the beads were dissolved in Dynal buffer A (PBS, pH 7.4, 2 mM EDTA, 5% BSA). Then, beads were equally divided into three fractions (Fig. 5f, step 4): *a*) saved as a control; *b*) incubated at 4 °C for 12 h with the lysate of EtOH-treated cells transiently transfected with both Rab6(T27N) plasmid and NMIIA siRNA; and *c*) incubated at 4 °C for 12 h with the lysate of EtOH-treated cells transiently transfected with Rab6(T27N) only. Therefore, fraction *b* was lacking both NMIIA-P-S1943 and endogenous Rab6a, while fraction *c* lacked only endogenous Rab6a (Fig. 5g, top panel-input and Supplementary Fig. S8). Finally, proteins of beads were separated on SDS PAGE on 4–15% gel (Bio-Rad) and subjected to W-B to detect giantin and Rab6a. Predictably, all three samples contained same level of giantin, however the Rab6a active protein was detected only in fractions *a* (control) and *b* (input lacking NMIIA-P-S1943) but not *c* (input contained NMIIA-P-S1943), indicating that Rab6a was released from the complex with giantin by NMIIA-P-S1943 (Fig. 5g, lower panel and Supplementary Fig. S8).

Also, we have observed the following phenomenon: in the Golgi fractions of control cells, the Rab6a dimer was detected^41^, but little, if any, Rab6a dimer was found at Golgi from either EtOH-treated VA-13 cells or hepatocytes from EtOH-fed rats (Fig. 5h and Supplementary Fig. S9). This surprising result underscores the importance of Rab6a dimerization for giantin function under normal circumstances^8^,^11^, and suggests that the dimeric form of both giantin and Rab6a are essential for maintaining compact Golgi morphology.

## Discussion

Hepatomegaly, or ballooning of the hepatocyte, and portal hypertension are some of the manifestations of alcoholic liver injury. Indeed, chronic alcohol administration induces an increase in liver weight due to accumulation of glycoproteins in the Golgi^20^. In recent years, we made significant progress in understanding the detailed molecular mechanisms of EtOH-induced intracellular alterations. We believe Golgi disorganization is one of the most crucial events that blocks transport of newly synthesized secretory or membrane glycoproteins from the Golgi to the basolateral membrane.

The results presented here, coupled with our previous observations, favor a scenario in which EtOH-induced Golgi disorganization is a multistep process. Disorganization is initiated by downregulation of Sar1a and subsequent deficiency of giantin, followed by an alteration of giantin and Rab6a complex and a progressive cooperation of phosphorylated NMIIA with Rab6a. The questions of which kinase catalyzes S1943 phosphorylation of NMIIA *in vivo* and how the activity of this kinase correlates with EtOH consumption seem most critical, and success in this area will help open many doors to prevention of alcohol metabolism’s damaging effects on the cell.

**Figure 6:**
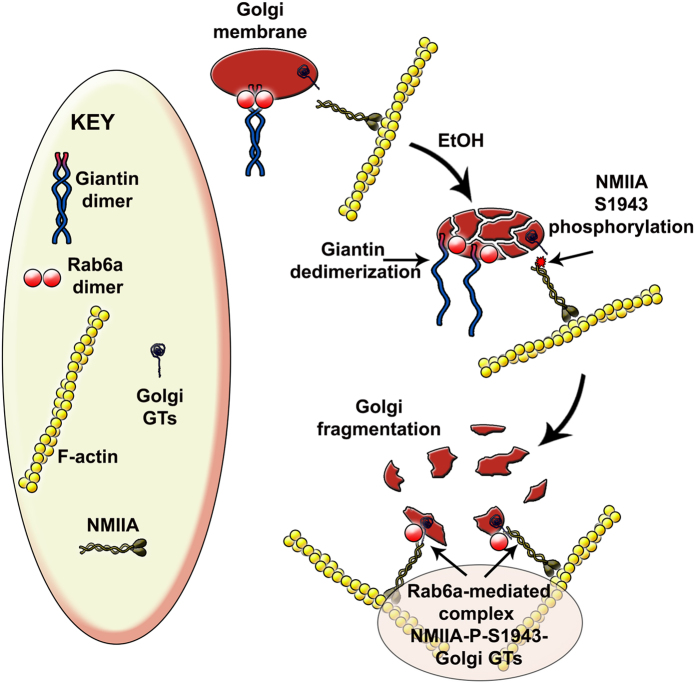
The proposed model of EtOH-induced and NMIIA-mediated Golgi disorganization in hepatocytes. Under normal conditions, Giantin dimerization maintains Golgi integrity. EtOH treatment induces dedimerization of both giantin^8^ and Rab6a and results in phosphorylation of NMIIA in S1943. NMIIA-P-S1943 competes with giantin for Rab6a GTPase and is recruited to the Golgi, likely through complex with the cytoplasmic tail of Golgi enzymes. The link NMIIA-Golgi enzymes provides a force for Golgi fragmentation.

The close relationship between Golgi matrix proteins and Rab GTPases proteins, on the one hand, and Rab GTPases and myosin proteins, on the other hand, is increasingly being viewed as essential for Golgi remodeling^42^,^43^. Here, for the first time we have shown that EtOH-induced Golgi fragmentation in hepatocytes is mediated by NMIIA. Most importantly, we have observed and experimentally proven the tug-of-war between giantin and NMIIA for Rab6a: the contribution of Rab6a in the recruitment of NMIIA at the Golgi becomes possible when Rab6a is released from the complex with giantin^17^. Therefore, Rab6a plays a dual role in the Golgi, serving as master regulator of Golgi organization and disorganization (Fig. 6).

We have revealed that NMIIA is tethered to the Golgi in S1943 phosphorylated form and uses Golgi GTs as the anchoring partner. Phosphorylation of S1943 in the NMIIA is one of the most widely observed posttranslational modifications detected for this isoform *in vivo*^44^. It has been shown previously that NMIIA rod domain does not bind to Golgi membranes when phosphorylated *in vitro* with CK II^35^. At the first glance the data appear to conflict with ours, however, we believe that this divergence mainly depends on the current understanding of the contribution of CK II in NMIIA S1943 phosphorylation. Indeed, a CK II consensus site on NMIIA (S1943) is highly phosphorylated in breast tumor cells during cell spreading and integrin-fibronectin engagement^45^; however, neither pharmacological nor siRNA KD of different CK II subunits significantly reduces S1943 phosphorylation, indicating that the CK II is probably not the *bona fide* kinase that regulates NMIIA S1943 phosphorylation *in vivo*. The role for S1943 phosphorylation seems critical for NMIIA involvement in the regulation of the migration of breast tumor cells, and during the epithelial to mesenchymal transition^39^,^46^. Given that breast tumor cells display disorganized Golgi phenotype^47^ and Golgi fragmentation is considered a potential hallmark of cancer progression^48^, it is an appealing idea that NMIIA-P-S1943 may play a grand role for not only cancer cell migration, but also for the activation of Golgi-associated downstream cascades that increase cell survival.

Our data provide a new insight into the understanding of the relationship between Golgi matrix and non-matrix proteins. We previously found that GTs employ different Golgi targeting mechanisms, using either giantin or GM130-GRASP65 as the primary docking sites^49^. Herein, we observed that NMIIA uses GTs as a partner to create a force for Golgi unstacking. This phenomenon highlights the importance of the cytoplasmic tail of GTs, not only for their Golgi retention^50^,^51^, but also for cooperation with motor proteins such as NMIIA; however, the details of interaction of GTs with the NMIIA need further rigorous study. Therefore, while matrix proteins primarily maintain Golgi organization^52^, residential enzymes are directly involved in Golgi remodeling, and this is confirmed by our current story.

We previously found in hepatocytes that EtOH treatment alters the generation of COPII vesicles through the suppression of an active, GTP-bound form of Sar1a^8^. Moreover, ultrastructural analysis revealed that depletion of Sar1a mimics EtOH effects: Golgi was similarly disrupted in swollen membranes and the level of giantin dimer was reduced via disrupted Golgi delivery of PDIA3. It is also interesting to note that EtOH-induced Golgi fission has no effect on ER-to-Golgi transportation of plasma membrane proteins, for example, the hepatic asialoglycoprotein receptor (ASGP-R); however, Golgi disorganization results in its sequestration in *cis-medial-*, but not *trans-*Golgi. Thus, alcohol-induced deficiency in COPII vesicle formation predetermines NMIIA-mediated Golgi fragmentation, which, in turn, compromises the Golgi-to-plasma membrane transportation of ASGP-R. We were also intrigued to note here, in addition to our previous observations, that co-silencing of Sar1a and NMIIA prevents Golgi disorganization, demonstrating that Sar1a KD-caused Golgi fragmentation is triggered by the action of NMIIA. In addition, similar to EtOH treatment, Sar1a KD induces segregation of Rab6a and giantin, confirming that impairment of COPII in hepatocytes is the cause of Golgi disorganization. We were also surprised to detect that in EtOH-treated samples, the amount of Rab6a dimer was significantly reduced in the Golgi fraction. This phenomenon indicates that Rab6a interacts with giantin in its dimer form, while in fragmented Golgi stacks (induced by EtOH treatment) Rab6a is present mostly as a monomer. This, coupled with previous observations, suggests that giantin and NMIIA compete for binding to Rab6a, and in the absence of the giantin dimer, Rab6a recruits NMIIA to the Golgi membranes^11^.

Thus, the actomyosin complex is required for EtOH-induced Golgi disorganization, and Rab6a is the GTPase which coordinates this process. Given that Rab6a is also widely present in the *trans*-Golgi and involved in intra-Golgi transportation and secretion of proteins^53^,^54^, for the future it will be reasonable to examine whether a connection exists between the two EtOH treatment-related phenomena: (a) interruption of ASGP-R trafficking to the *trans*-Golgi and (b) Rab6a dedimerization in the Golgi. Finally, whether prevention of NMIIA S1943 phosphorylation restores ASGP-R distribution in both human hepatocytes and animal liver cells will, of course, be the logical focus of our future studies.

## Methods

### Antibodies and reagents

The primary antibodies used were: a) rabbit polyclonal – NMIIA (Sigma), phospho-NMIIA (Ser1943) (Cell Signaling Technology), giantin, Man-II, and Rab6a (Abcam); b) mouse monoclonal - Rab6a (Santa Cruz Biotechnology), β-actin (Sigma), Sar1a, giantin (Abcam); c) mouse polyclonal - ST3Gal1 (Abnova). The secondary antibodies (Jackson ImmunoResearch) were: a) HRP-conjugated donkey anti-rabbit and donkey anti-mouse for Western-blotting; b) donkey anti-mouse Alexa Fluor 488 and anti-rabbit Alexa Fluor 594 for immunofluorescence. Blebbistatin (Sigma) and latrunculin A (Tocris Bioscience) were dissolved in dimethyl sulfoxide (DMSO) immediately before use. Cells treated with a corresponding concentration of DMSO served as controls. The regular working concentrations were: blebbistatin −25 μM for up to 24 h; latrunculin A −0.2 μM for up to 24 h. Active human Rab6a full length protein was obtained from Abcam.

### Cell culture and EtOH administration

HepG2 cells, obtained from ATCC (Rockville, MD), were grown in Dulbecco’s modified Eagle medium (DMEM) as described earlier^24^. HepG2 cells transfected with mouse ADH1 (VA-13 cells) were obtained from Dr. Dahn Clemens at the Department of Veterans Affairs, Nebraska Western Iowa HCS^24^. Twenty-four hours after seeding cells (at ∼75% confluence), culture media were replaced with another containing 35 mM EtOH for an additional 72 h. The conditioned medium was replaced every 12 h to maintain same EtOH concentration. Control cells were seeded at the same time as treated cells and maintained on same medium without EtOH.

Primary rat hepatocytes from control and EtOH-fed animals were prepared from male Wistar rats. Rats weighing 140–160 g were purchased from Charles River Laboratories. Initially, the animals were fed a Purina chow diet and allowed to acclimate to their surroundings for a period of 3 days. Then the rats were paired according to weight and fed either control or EtOH containing (36% of calories) Lieber-DeCarli diet for periods of 5–7 weeks (Dyets, Inc). This protocol was approved by the Institutional Animal Care and Use Committee of the Department of Veterans Affairs, Nebraska Western Iowa HCS, and the University of Nebraska Medical Center. The animals were handled in accordance with applicable local and federal regulations concerning laboratory animals. Hepatocytes were obtained from the livers of control and EtOH-fed rats by a modified collagenase perfusion technique previously described by Casey *et al.*^55^. The isolated cells were washed with Krebs-Ringer buffer, purified over a 35% Percoll gradient, and equilibrated at 37° for 45 min in William’s buffer containing 0.1% BSA. Hepatocytes were washed, counted, re-suspended in William’s/BSA medium, and placed on ice until they were divided for the various experiments.

### Immunoprecipitation (IP) and transfection

For identification of proteins in the complexes pulled down by IP, confluent cells grown in a T75 flask were washed three times with 6 ml PBS each, harvested by trypsinization, and neutralized with soybean trypsin inhibitor at a 2× weight of trypsin. The IP steps were performed using the Pierce Co-Immunoprecipitation Kit (Thermo Scientific) according to manufacturer’s instructions. Mouse and rabbit non-specific IgG were used as a non-specific controls. All cell lysate samples for IP experiments were normalized by appropriate proteins. To determine whether the target protein was loaded evenly, the input samples were preliminarily run on a separate gel with different dilutions of control samples vs. treated, then probed with anti-target protein Abs. The intensity of obtained bands was analyzed by ImageJ software, and samples with identical intensity were subjected to IP. *MYH9* (myosin, heavy polypeptide 9, non-muscle, NMIIA), *SAR1A* and scrambled on-targetplus smartpool siRNAs were purchased from Santa Cruz Biotechnology. All products were consisted of pools of three target-specific siRNAs. Cells were transfected with 100 nM siRNAs using Lipofectamine RNAi MAX reagent (Life science technologies). PCMV-intron myc Rab6 T27N was a gift from Terry Hebert (Addgene plasmid # 46782)^56^, and pCMV-eGFP-NMHC-IIAΔtailpiece was a gift from Tom Egelhoff (Addgene plasmid # 35689)^57^. Transient transfection of VA-13 cells was carried out using the Lipofectamine 2000 (Life Science technologies) following the manufacturer’s protocol.

### Phosphorylation assay

Cell samples were re-suspended in an EDTA-free CIP buffer, containing 100 mM NaCl, 50 mM Tris-HCl, 10 mM MgCl_2_, and 1 mM DTT (New England Biolabs). Samples were further treated with CIP (New England Biolabs) (1 unit CIP per microgram of protein) in the absence or presence of a phosphatase inhibitor, β-glycerophosphate (50 mM) for 60 min at 37 °C. Then, samples were analyzed by SDS PAGE followed by W-B.

### Isolation of NMIIA-P-S1943 from VA-13 cell lysates via biotinylated N-terminal peptides of glycosyltransferases

The following N-terminal biotinylated peptides representing CTs of human GTs were obtained from the GenicBio BioTech (Shanghai, China): core 2N-acetylglucosaminyltransferase-1 (C2GnT1) (Biotin-MLRTLLRRRL), β-galactoside α-2,3-sialyltransferase-1 (ST3Gal1) (Biotin-AGSMVTLRKRTLKV), β1,3-N-acetylglucosaminyltransferase 6 (core 3 synthase) (Biotin-AGSMAFPCRRSLTAKTL), α-mannosidase II (Man-II) (Biotin-AGSMKLSRQ), polypeptide N-acetylgalactosaminyltransferase 2 (ppGalNAcT2) (Biotin-AGSMRRRSRM). Control peptide Biotin-GHGTGSTGSGSMLRTLLRRRL was purchased from LifeTein LLC (South Plainfield, NJ). To isolate NMIIA-P-S1943, 20 μl of peptides in 25% acetic acid (0.1 mg/ml) was mixed with 20–40 μl of cell lysate (1.5–3.5 mg/ml of protein). After incubation at 37 °C for 1 h, 100 μl of Dynabeads M-280 Streptavidin (Dynal, Norway) was added. Following gentle rotation for additional 30 min, the beads with immobilized complexes were trapped with a magnet. The captured proteins were separated on 8% SDS-PAGE followed by Western blotting with anti-NMIIA-P-S1943 Ab. Control peptide was used by the same procedure, and another sample of cell lysate incubated with an appropriate amount of 25% acetic acid and followed by treatment with Dynabeads served as a control.

### Confocal immunofluorescence microscopy

Staining of cells was performed by the methods described previously^15^. Slides were examined under a Zeiss 510 Meta Confocal Laser Scanning Microscope and LSM 800 Zeiss Airyscan microscope performed at the Advanced Microscopy Core Facility of the University of Nebraska Medical Center. Images were analyzed using ZEN 2009 software. For some figures, image analysis was performed using Adobe Photoshop and ImageJ. F-actin staining was performed using CytoPainter F-actin Staining Kit-Blue Fluorescence according to the manufacture’s (Abcam) protocol. Statistical analysis of colocalization was performed by ImageJ, calculating the Pearson correlation coefficient^58^.

### Three-dimensional structured illumination (3D-SIM) microscopy and image analysis

SIM imaging of Golgi ribbons was performed on a Zeiss ELYRA PS.1 super resolution scope (Carl Zeiss Microscopy, Germany) using a PCO.Edge 5.5 camera equipped with a Plan-Apochromat 63 × 1.4 oil objective. Optimal grid sizes for each wavelength were chosen according to the recommendations of the manufacturer. For 3D-SIM, stacks with a step size of 110 nm were acquired sequentially for each fluorophore, and each fluorescent channel was imaged with three pattern rotations with 3 translational shifts. The final SIM image was created using modules build into the Zen Black software suite that accompanies the imaging setup. Analyses were undertaken on 3D-SIM data sets in 3D using IMARIS versions 7.2.2–7.6.0 (Bitplane Scientific). The 3D mask was obtained by applying a Gaussian filter to merged channels, thresholding to remove low-intensity signals, and converting the obtained stack into a binary file that mapped all voxels of interest for coefficient calculation. For colocalization studies, IMARIS “Colocalization Module” was used. To avoid subjectivity all thresholds were automatically determined using algorithms based on those of Costes *et al.*^59^, which is based on the exclusion of intensity pairs that exhibit no correlation. Colocalization was determined by Pearson’s coefficient, which represents a correlation of channels inside the colocalized regions. After calculation, the colocalization pixels were displayed as white. 3D animation was also generated using IMARIS “Animation Module.”

### *In situ* Proximity Ligation Assay

The assay was performed using the Duolink kit (Olink Bioscience) according to the manufacturer’s protocol. Briefly, VA-13 cells were treated with two different combination of Abs: (a) mouse anti-giantin and rabbit anti-Rab6a, and (b) mouse anti-Rab6a and rabbit anti-NMIIA-P-S1943, then with oligonucleotide-conjugated anti-mouse minus and anti-rabbit plus proximity ligation assay secondary probes. The oligonucleotides were used to generate circular DNA, which was then amplified and tagged with a red fluorescence dye. Following DAPI staining of the nuclei, the cells were examined by confocal fluorescence microscopy. The red spots, which represent interaction, from 60 randomly selected images were counted and plotted.

### Isolation of Golgi membrane fractions by sucrose gradient centrifugation

Golgi membrane fractions were isolated using methods described previously^15^. Cells from ten-to-twelve 75 cm^2^ cell culture flasks were harvested with PBS containing 0.5× protease inhibitors (1.2 ml per flask). Then, after centrifugation for 5 min at 1000 rpm and 4 °C, the pellet was resuspended in 3 ml of homogenization buffer (0.25 M sucrose, 3 mM imidazole, 1 mM Tris-HCl; pH7.4, 1 mM EDTA). The cells were homogenized by drawing ~ 30 times through a 25-gauge needle until the ratio between unbroken cells and free nuclei becomes 20%:80%. The postnuclear supernatant was obtained by centrifugation at 2,500 rpm and 4 °C for 3 min and then the supernatant was adjusted to 1.4 M sucrose by the addition of ice-cold 2.3 M sucrose in 10 mM Tris-HCl (pH 7.4). Next, 1.2 ml of 2.3 M sucrose at the bottom of tube was overlaid with 1.2 ml of the supernatant adjusted to 1.4 M sucrose followed by sequential overlay with 1.2 ml of 1.2 M and 0.5 ml of 0.8 M sucrose (10 mM Tris-HCl, pH 7.4). The gradients were centrifuged for 3 h at 38,000 rpm (4 °C) in a SW40 rotor (Beckman Coulter). The turbid band at the 0.8 M/1.2 M sucrose interface containing Golgi membranes was harvested in ~500 μl aliquot by syringe puncture. The fraction at concentration of ~1.0–1.4 mg protein/ml was used for the experiments mentioned in the Results section.

### RT-PCR analysis of gene expression

RNA from cultured VA-13 cells was isolated by TRI-REAGENT (MRC Inc.) according to the manufacturer’s instruction. To prepare cDNA, 2 μg RNA was used in a 20 μl reaction mixture using a Verso reverse transcriptase kit (Thermo scientific) as follows: 5 min at RT, 60 min at 42 °C, and 2 min at 95 °C. Quantitative real-time PCR was performed in 10 μl reaction volume in a 96-well plate, using 2 μl of diluted (1:1) cDNA with SYBR^®^ Premix Ex Taq™ (Takar Bio Inc.) on a Mastercycler Epgradient realplex (Eppendorf). The PCR conditions included 1 cycle at 95 °C for 2 min, followed by 45 cycles at 95 °C for 15 s, 60 °C for 15 s, and 72 °C for 45 s. The data were analyzed using Eppendorf realplex software, version 1.5 (Eppendorf). Glyceraldehyde-3-phosphate dehydrogenase (GAPDH) was included for every sample as the control reaction. Relative fold differences in transcript expression were determined using the following comparative CT method: 2^−[ΔCt (Target)]^ ×100 where ΔCt = Ct(Target) − Ct (GAPDH) as described previously^60^. The results were expressed as the fold relative to that (100%, 1 fold) of GAPDH.

## Additional Information

**How to cite this article**: Petrosyan, A. *et al.* The role of Rab6a and phosphorylation of non-muscle myosin IIA tailpiece in alcohol-induced Golgi disorganization. *Sci. Rep.* **6**, 31962; doi: 10.1038/srep31962 (2016).

## Supplementary Material

Supplementary Information

Supplementary Movie S1

Supplementary Movie S2

Supplementary Movie S3

Supplementary Movie S4
