# Supplementary material for: The role of Rab6a and phosphorylation of non-muscle myosin IIA tailpiece in alcohol-induced Golgi disorganization
Source: Sci Rep. 2016 Aug 18;6:31962. doi: 10.1038/srep31962 (PMC4989220; doi:10.1038/srep31962)
Supplement: Supplementary Information [file srep31962-s1.pdf]

Supplemental materials

The role of Rab6a and phosphorylation of non-muscle myosin IIA tailpiece in alcohol-induced Golgi disorganization

Armen Petrosyan, Carol A. Casey and Pi-Wan Cheng

Figure S1

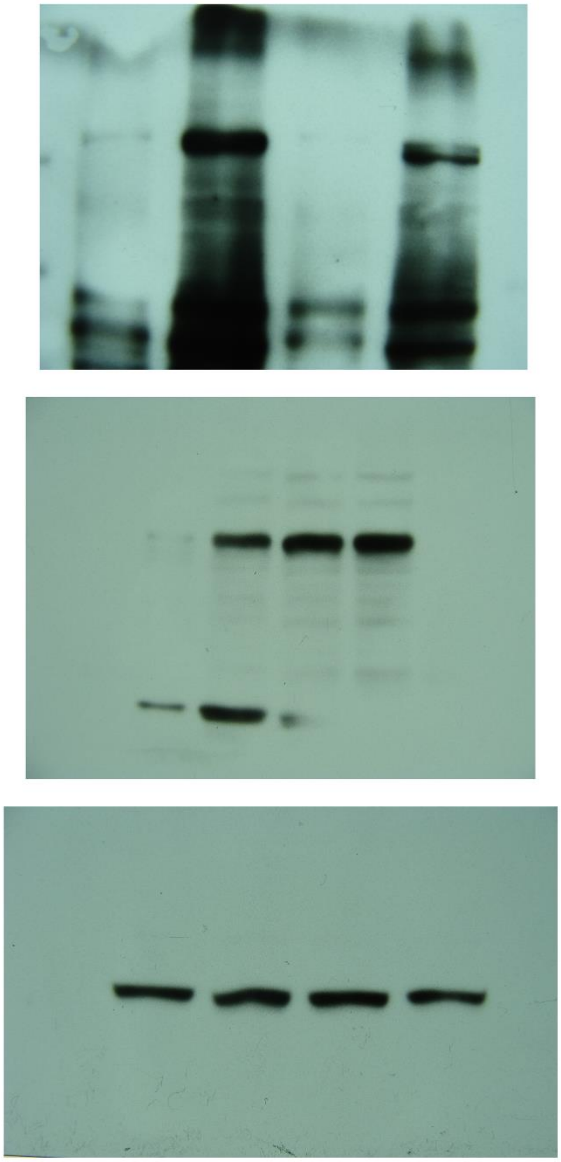

Figure S1. The full-length gels blots that included in Figure 3f.

Figure S2

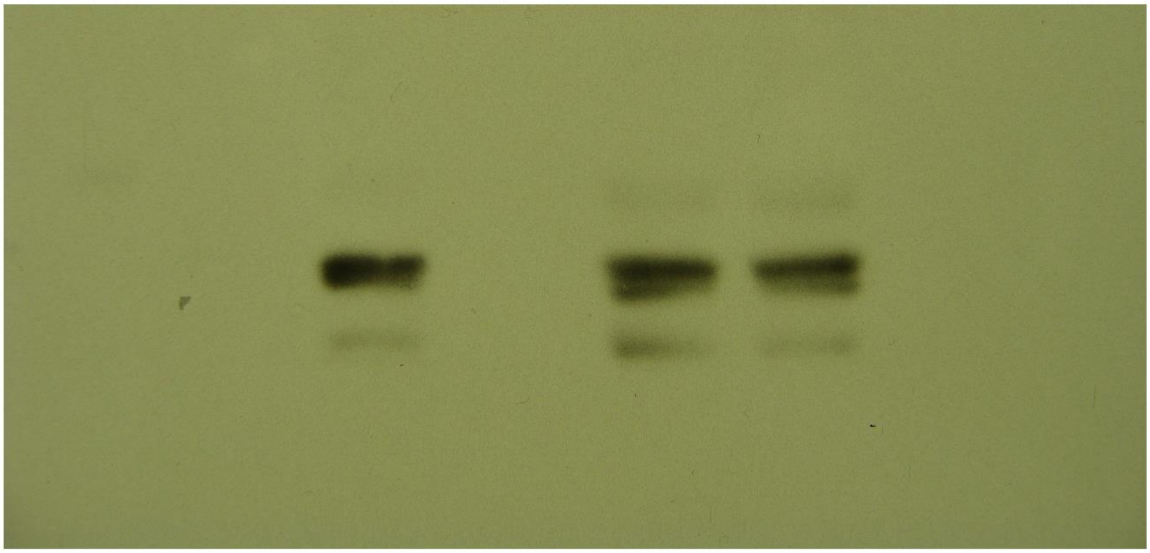

Figure S2. The full-length gels blots that included in Figure 3h.

Figure S3

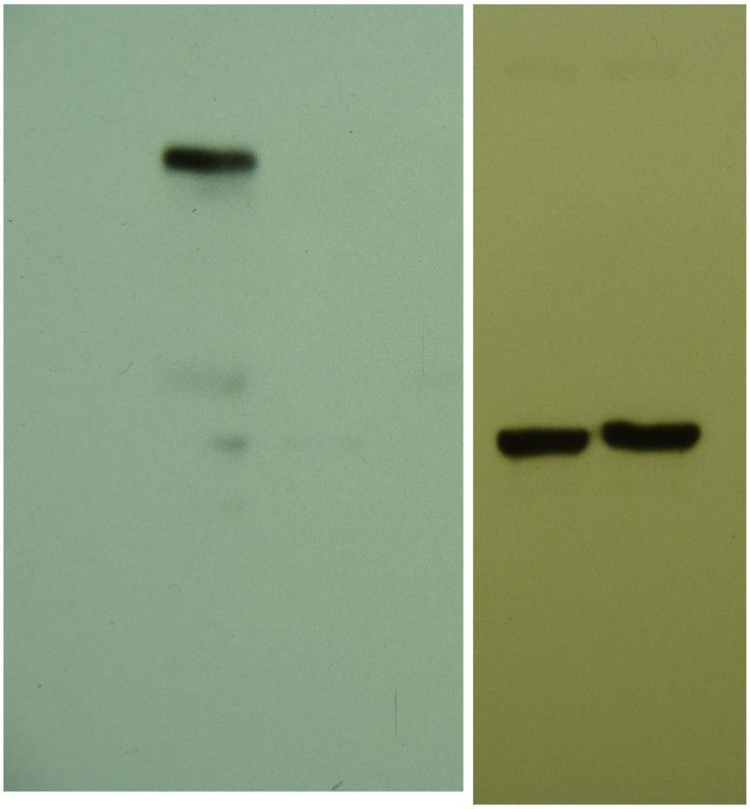

Figure S3. The full-length gels blots that included in Figure 3i.

Figure S4

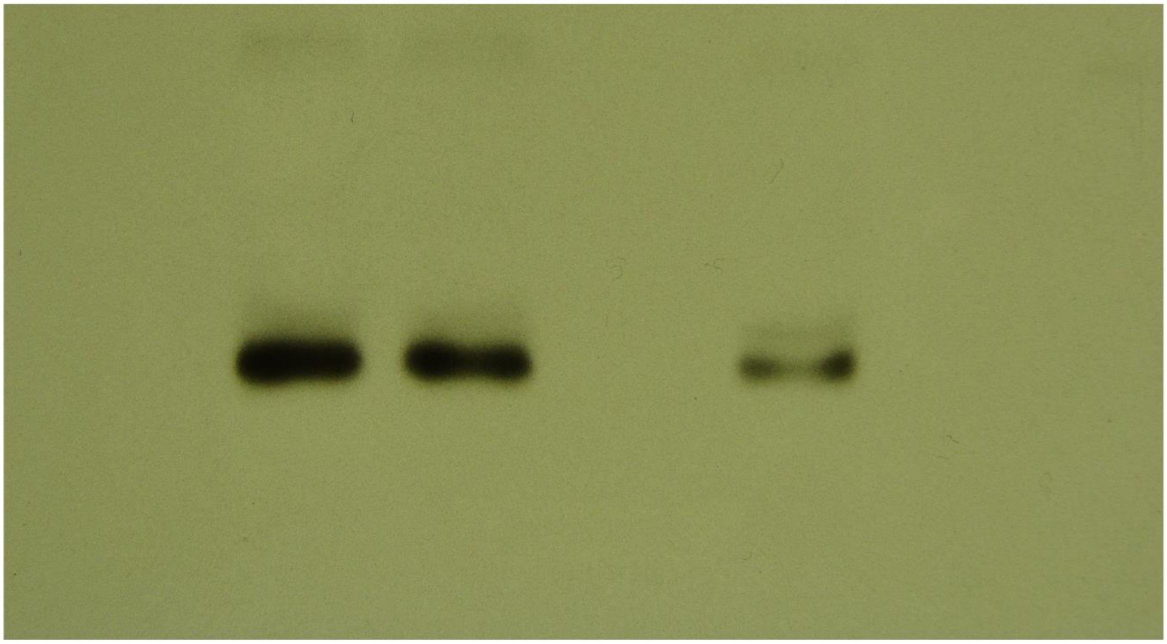

Figure S4. The full-length gels blots that included in Figure 3j.

Figure S5

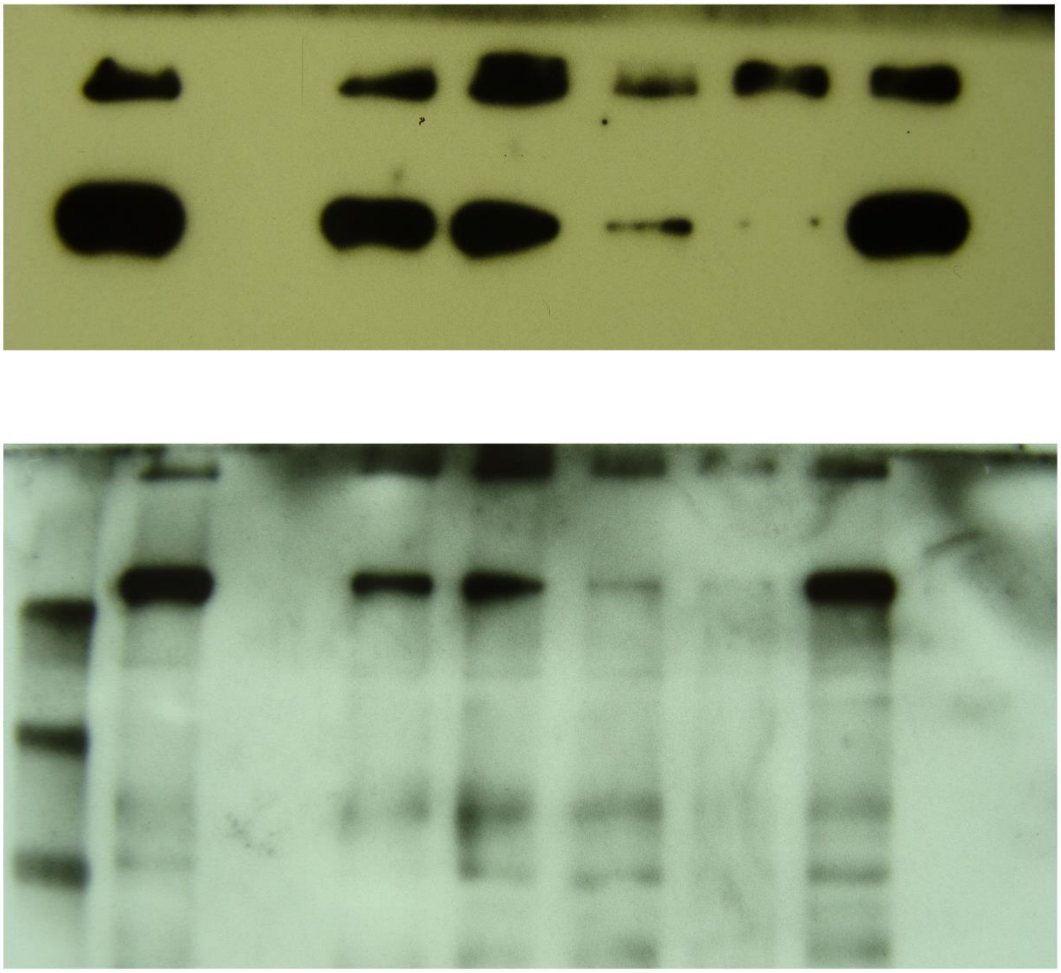

Figure S5. The full-length gels blots that included in Figure 4a.

Figure S6

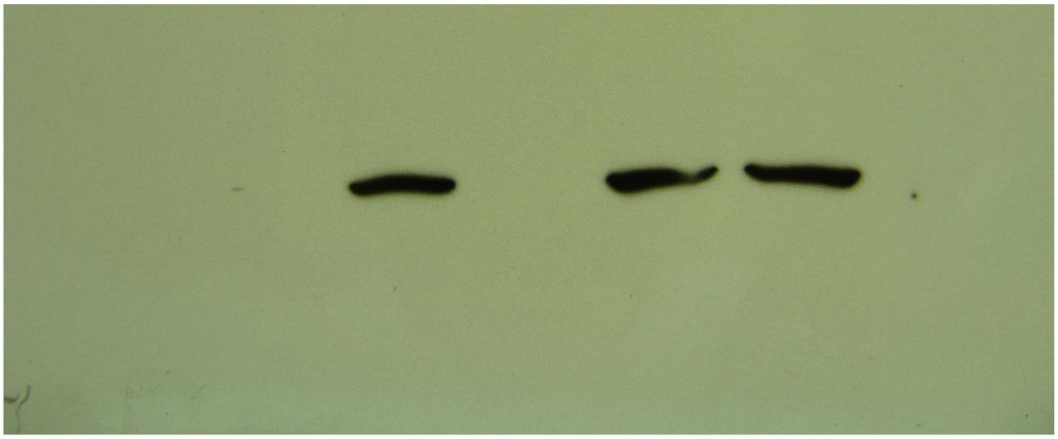

Figure S6. The full-length gels blots that included in Figure 4c.

Figure S7

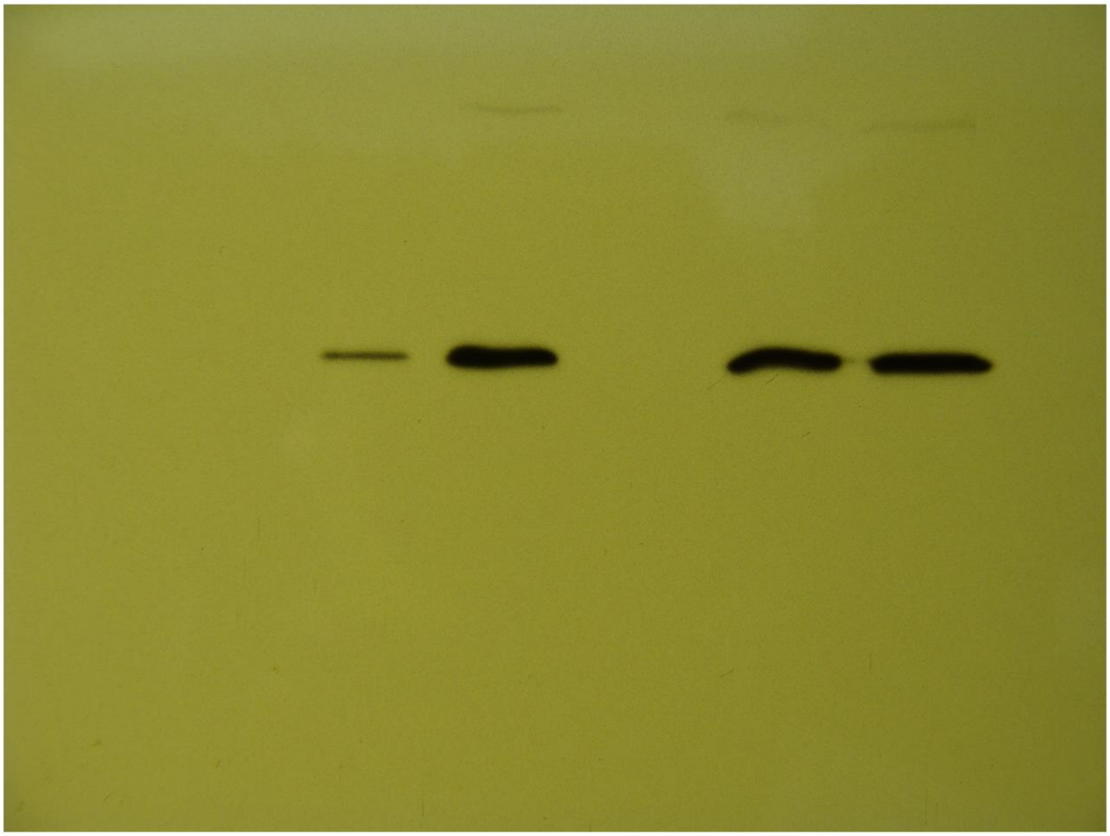

Figure S7. The full-length gels blots that included in Figure 4f.

Figure S8

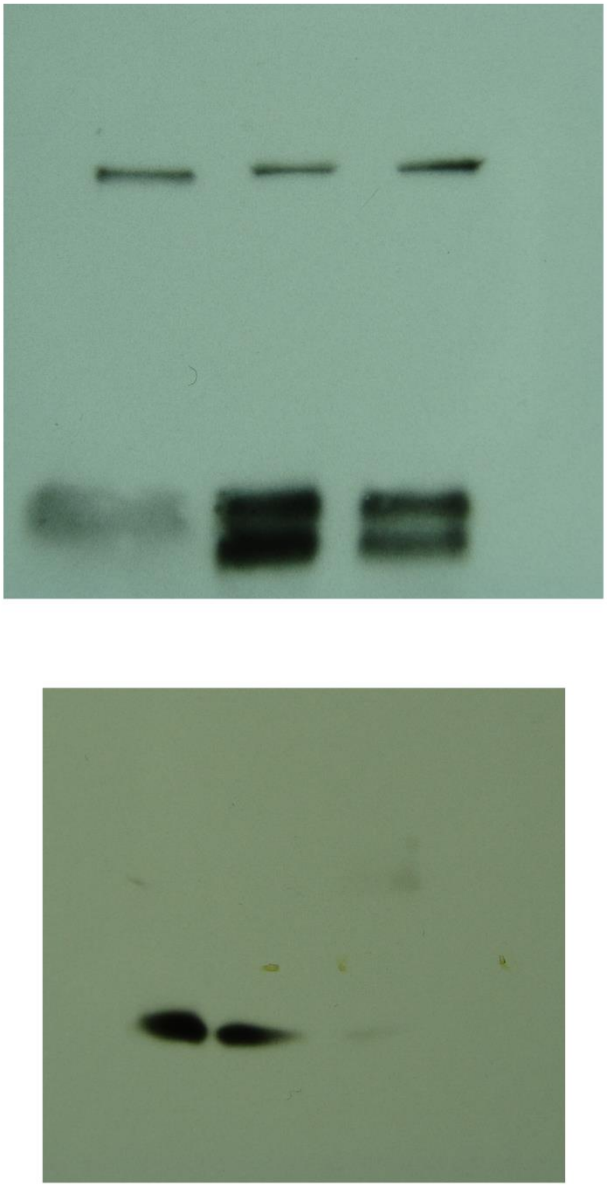

Figure S8. The full-length gels blots that included in Figure 5g.

Figure S9

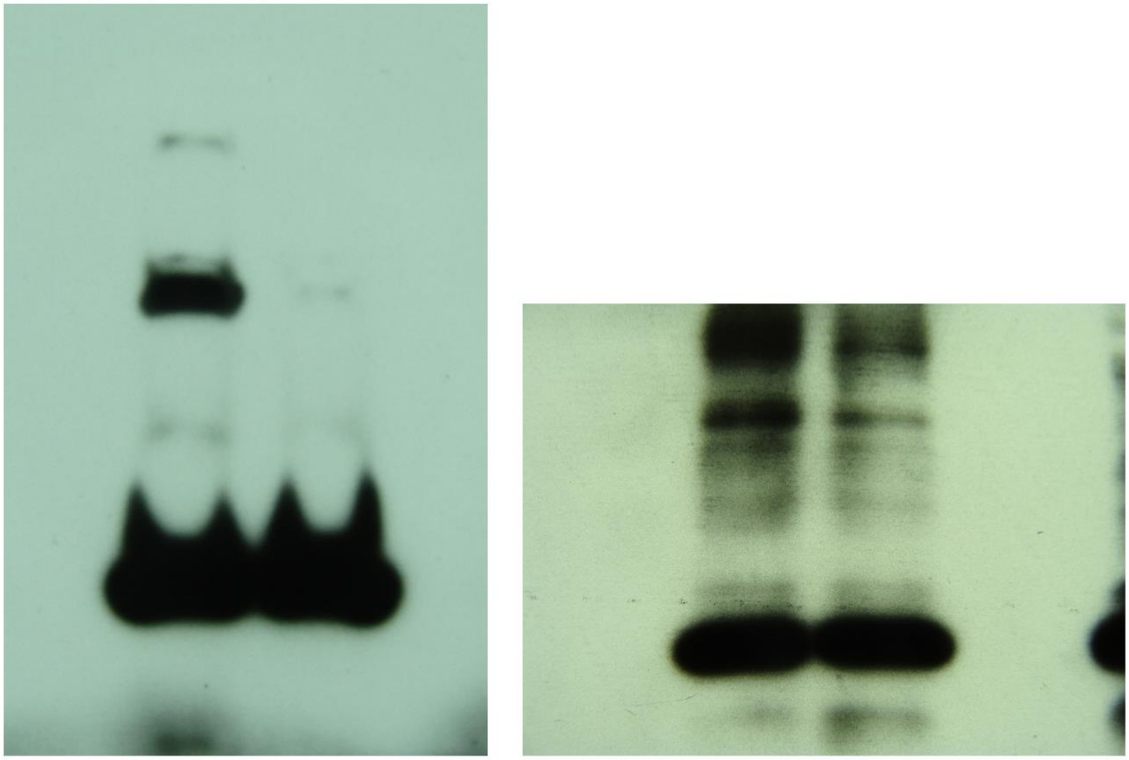

Figure S9. The full-length gels blots that included in Figure 5h.

Legends for the Supplementary Movies

Supplementary Movie S1. 3D SIM animation of control VA-13 cell co-stained for ST3Gal1 (green) and NMIIA-P-S1943 (red).

Supplementary Movie S2. 3D SIM animation of VA-13 cell treated with 35 mM EtOH and co-stained for ST3Gal1 (green) and NMIIA-P-S1943 (red).

Supplementary Movie S3. 3D SIM animation of control rat hepatocyte co-stained for Man-II (green) and NMIIA-P-S1943 (red).

Supplementary Movie S4. 3D SIM animation of hepatocyte from EtOH-fed rat co-stained for Man-II (green) and NMIIA-P-S1943 (red).
